# Music reduces patient-reported pain and anxiety and should be routinely offered during flexible cystoscopy: Outcomes of a systematic review

**DOI:** 10.1080/2090598X.2021.1894814

**Published:** 2021-03-03

**Authors:** Anusha Gauba, Meghana N. Ramachandra, Mansi Saraogi, Robert Geraghty, B.M. Zeeshan Hameed, Omar Abumarzouk, Bhaskar K. Somani

**Affiliations:** a Department of Urology, University Hospital Southampton NHS Trust, Southampton, UK; b Department of Urology, Kasturba Medical College Manipal, Manipal Academy of Higher Education, Manipal, India; c Department of Surgery, Hamad General Hospital, Hamad Medical Corporation, Doha, Qatar

**Keywords:** Music, complementary therapy, cystoscopy, analgesia, pain, anxiety

## Abstract

**Objective**: To conduct a systematic review of the literature to assess whether music reduces the use of analgesics and anxiolytics during flexible cystoscopy.

**Methods**: The systematic review was performed in line with the Cochrane guidelines and Preferred Reporting Items for Systematic Reviews and Meta-analyses (PRISMA) checklist. The databases searched included the Medical Literature Analysis and Retrieval System Online (MEDLINE), Scopus, Cumulative Index to Nursing and Allied Health Literature (CINAHL), Clinicaltrials.gov, the Excerpta Medica dataBASE (EMBASE), Cochrane library, Google Scholar, and Web of Science from inception of the databases to February 2020. The primary outcome measure was the effect of music on pain and anxiety, and secondary outcome measures were patient heart rate and blood pressure.

**Results**: The initial search yielded 234 articles and after going through titles and abstracts, four studies (399 patients, 199 in the music group and 200 in no music group) were included for the final review. There were three randomised controlled trials and one prospective study published between 2014 and 2017. These studies were done in China, the USA and Italy, with the study duration between 9 and 24 months. All patients had 2% topical lignocaine jelly given per-urethra before the procedure. The choice of music was classical in three studies and a mixture of different music types in one study. Three of the four studies showed significantly reduced pain and anxiety with the use of music for flexible cystoscopy procedures. Heart rate was noted to be higher for the no music group, reflecting a higher pain perceived by these patients.

**Conclusion**: The present review showed that listening to music was associated with reduced anxiety and pain during flexible cystoscopy. Listening to music is therefore likely to increase procedural satisfaction and willingness to undergo the procedure again, considering repeated flexible cystoscopy is often needed for surveillance. As music is simple, inexpensive and easily accessible, it should be routinely offered to patients for outpatient and office-based urological procedures.

**Abbreviations**: IQR: interquartile range; NRS: numerical rating scale; PTSD: post-traumatic stress disorder; RCT: randomised control trial; STAI: State–trait Anxiety Inventory; VAS: visual analogue scale

## Introduction

Flexible cystoscopy is performed widely for diagnostic, surveillance, and therapeutic purposes. It is the preferred alternate to rigid cystoscopy, as it is less painful and the latter usually requires general anaesthesia [1,2]. However, it can still cause anxiety and pain, especially in male patients [3]. Over the last few years technological and pharmacological innovations have helped reduced pain and patient anxiety during various urological procedures. Music has also been advocated for various procedures including lithotripsy, prostate biopsy, percutaneous nephrostomy, and urodynamic studies [4,5].

Music reduces discomfort by activating the cingulo-frontal cortex and pain modulation [6]. It is safe, inexpensive and has been proven to be effective in other urological office-based procedures. Other complementary therapies used to improve the patient experience are audio-visual distraction, acupressure, acupuncture, and transcutaneous electrical nerve stimulation (TENS) [5]. While flexible cystoscopy is now the standard investigation of choice for haematuria, LUTS and surveillance of bladder cancer, few studies have looked at the role of music in reducing pain and anxiety for this procedure [7–14].

We aimed to conduct a systematic review of literature to assess the effect of music on flexible cystoscopy, and if this reduced the use of analgesics and anxiolytics. Our secondary aim was to look at the effect of music on patient parameters such as heart rate and blood pressure.

## Methods

### Study population

Population: Adults undergoing flexible cystoscopy under local anaesthesia.

Intervention: Music.

Comparator: No music.

Outcome: Results (analgesia, anxiety), patient parameters (heart rate, blood pressure)

Study design: Systematic review

Inclusion criteria:

1. Randomised control trials (RCTs) and prospective studies in English language reporting on adults undergoing flexible cystoscopy.
2. Procedure under local anaesthesia.

Exclusion criteria:

1. Non-English language articles.
2. Review articles or case reports.
3. Grey literature and studies where outcomes of interest were not presented.

### Search strategy and selection criteria

The systematic review was performed in line with the Cochrane Collaboration guidelines and Preferred Reporting Items for Systematic Reviews and Meta-analyses (PRISMA) checklist. The databases searched included the Medical Literature Analysis and Retrieval System Online (MEDLINE), Scopus, Cumulative Index to Nursing and Allied Health Literature (CINAHL), Clinicaltrials.gov, the Excerpta Medica dataBASE (EMBASE), Cochrane library, Google Scholar, and Web of Science from inception of databases to February 2020. The search terms included but were not limited to ‘music’, ‘sound’, ‘complementary therapy’, ‘flexible cystoscopy’, ‘outpatient’ and ‘complementary medicine’. These terms were combined using the Boolean operators ‘AND’ and ‘OR’ to refine the search. Two reviewers (A.G. and M.R.) independently identified all studies that fitted our inclusion criteria for the review and discrepancies were resolved by consensus with the senior author (B.K.S.).

### Data extraction and analysis

The primary outcome measures were the effect of music on pain (measured using a visual analogue scale [VAS]) and anxiety (using the State–trait Anxiety Inventory [STAI] or delta STAI anxiety score (post- minus pre-anxiety score) or pain intensity numerical rating scale [NRS]). Secondary outcome measures were patient heart rate and blood pressure. Information was also collected on study design, patient demographics, year of publication, type of music, delivery method, VAS and STAI scores, and effect on vital parameters such as heart rate and blood pressure. Data were collected using Microsoft Excel®, version 19.0 (Microsoft, Redmond, WA, USA). Statistical analysis and forest plot generation using ‘metafor’ package in R (R Foundation for Statistical Computing, Vienna, Austria). Statistical heterogeneity was tested for using *I*^2^, Tau^2^ and Cochran’s *Q*. A *P *< 0.05 was considered statistically significant. The *I*^2^ values were interpreted according to Chapter 9.5.2 of the Cochrane Handbook.

## Results

The initial search yielded 234 articles and after rigorous scrutiny of titles and abstracts, only four studies (399 patients, 199 in the music group and 200 in no music group) were included for the final review [7,10,11,14]. It included three randomised controlled trials (RCTs) and one prospective study published between 2014 and 2017 (Tables 1–3).

**Table 1.** Characteristics of the four included studies

Reference	Duration of RCT, months	Country	Total number of patients Music/no music	Type of music	Patient selection/delivery	Duration of music, min	Score systems	Analgesia used (Pre-cystoscopy dwell time, min)	Antidepressant/anti-anxiety medications music/no music, *n*	Size of cystoscope, F
Zhang *et al*., 2014 [7]	12	China	124 62/62	Classical/ Chinese folk/Popular/ Foreign	Yes/headphones	Procedure	VAS/STAI	Lidocaine 2% 10 mL (3)	NR	16
Raheem *et al*., 2015 [10]	24	USA	137 73/64	Classical	No/not known	15–20 (including 2% lidocaine gel dwell time)	VAS/STAI/delta STAI anxiety score	Lidocaine 2% 10 mL (15)	13/13	15
Mirheydar *et al*., 2015 [11]	NR	USA	38 14/24	Classical	No/NR	Procedure	VAS/STAI/delta STAI anxiety score	Lidocaine 2% 10 mL (10)	4/6	15
Falavolti *et al*., 2017 [14]	9	Italy	100 50/50	Classical	No/NR	15–20 (including 2% lidocaine gel dwell time)	VAS/NRS	Lidocaine 2% 10 mL (15)	NR	16

NR: not reported.

**Table 2.** Summary of the demographics of the four included studies

Reference	No. of patients music/no music	Age, years, mean (SD, range) music/no music	Sex, M:F, *n* music/no music	Race, *n* music/no music	Diagnostic cystoscopy, *n* music/no music	Surveillance cystoscopy, *n* music/no music
Zhang *et al*., 2014 [7]	62/62	64.8 (11.2, 33–84)/ 62.0 (12.7, 25–81)	Males	Not reported	Mean (SD) 36.5 (6.6)/41.0 (6.8)	Mean (SD) 33.4 (5.0)/38.3 (6.1)
Raheem *et al*., 2015 [10]	73/64	65.8 (9.9)/67.1 (10.4)	Both 70:3/59:5	Caucasian 56/47 Other 17/17	32/40	41/29
Mirheydar *et al*., 2015 [11]	14/24	67.1 (9.9)/65.3 (10.4)	Males	Caucasian 10/15 Other 4/9	12/8	12/14
Falavolti *et al*., 2017 [14]	50/50	68 (13)/71 (9)	Both	Not reported	17/21	33/29

**Table 3.** Summary of studies with recorded parameters

	Before procedure					After procedure					
Study	SBP, mmHg, mean (SD) music/no music	DBP, mmHg, mean (SD) music/no music	Heart rate, beats/min, mean (SD) music/no music	STAI anxiety score, music/no music	VAS pain score	SBP, mmHg, mean (SD) music/no music	DBP, mmHg, mean (SD) music/no music	Heart rate, beats/min, mean (SD) music/no music	STAI anxiety score, music/no music	VAS pain score, music/no music	Delta STAI anxiety score, music/no music
Zhang *et al*., 2014 [7]	NR	NR	72.6 (7.7)/72.4 (7.1)	Mean (SD) 41.6 (7.9)/41.4 (7.6)	NR	NR	NR	76.0 (7.3)/79.8 (5.5)	Mean (SD) 76.0 (7.3)/79.8 (5.5)	Mean (SD) 1.63 (1.09)/2.53 (1.34)	NR
Raheem *et al*., 2015 [10]	135.5 (17.7)/135.5 (17.9)	79.4 (12.9)/80.0 (11.5)	73.5 (13.3)/75.3 (14.3)	Median (IQR) 31(25–41)/ 35(25–45)	NR	139.1 (22.8)/137.2 (17.3)	81.8 (13.0)/83.0 (11.6)	71.3 (15.6)/74.9 (14.2)	Median (IQR) 30 (23–39)/35 (28–49)	Median (IQR) 0 (0–1)/ 2 (1–2)	Median (IQR) 0 (–3 to 0)/ 2 (0–4)
Mirheydar *et al*., 2015 [11]	135.5 (17.7)/135.5 (17.9)	79.4 (12.9)/80.0 (11.5)	73.5 (13.3)/75.3 (14.3)	NR	NR	139.1 (22.8)/137.2 (17.3)	81.8 (13.0)/83.0 (11.6)	71.3 (15.6)/ 74.9 (14.2)	Median (IQR) 30 (23–39)/35 (28–49)	Median 1.6/1.5	Median 1.46/0.78
Falavolti *et al*., 2017 [14]	MAP 108 (13)/109(11)	–	–	–	–	MAP 107 (14)/108 (12)	–	No statistical difference	–	76%/28% VAS 2	–

DBP: diastolic blood pressure; MAP: mean arterial pressure; NR: not reported; SBP, systolic blood pressure.

These studies were done in China, the USA and Italy, with the study duration between 9 and 24 months. All patients had 2% topical lignocaine jelly given per-urethra before the procedure. The choice of music was classical in three studies and a mixture of different music types in one study. Three of the four studies showed significantly reduced pain and anxiety with the use of music for flexible cystoscopy procedures.

### Meta-analysis

**Figure 1.**
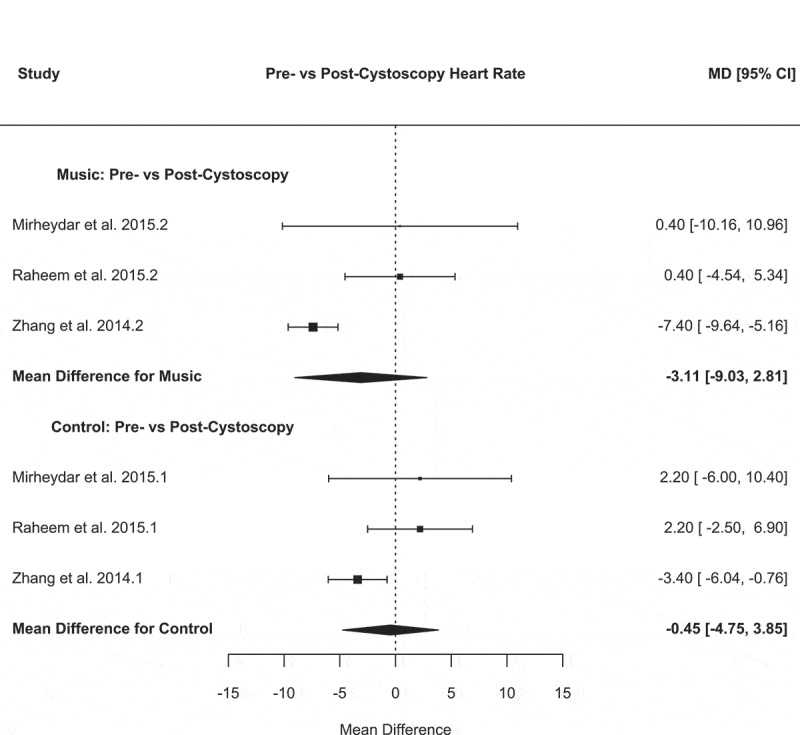
Forest plot for pre- vs post-cystoscopy heart rate

**Figure 2.**
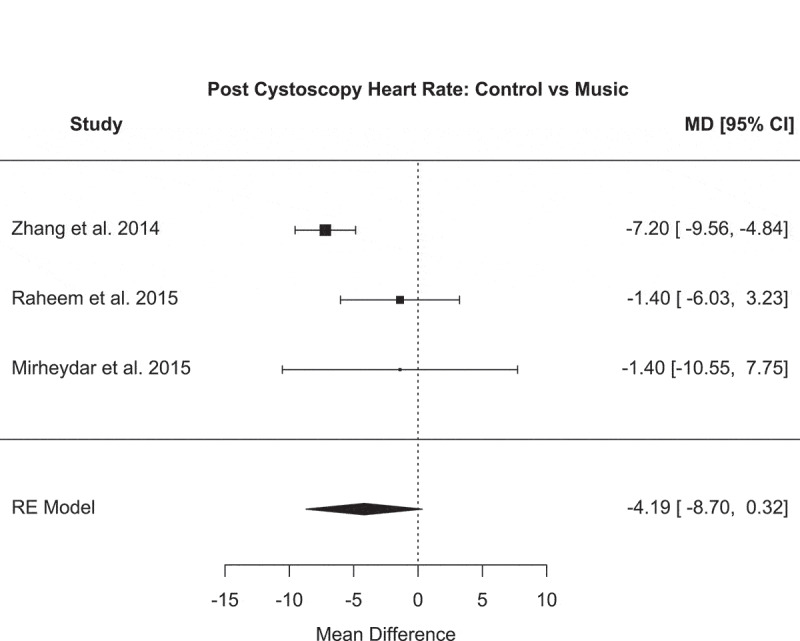
Forest plot for post-cystoscopy heart rate: control vs music

Heart rate was the only variable that was consistently reported. Therefore, a meta-analysis of heart rate (before vs after cystoscopy), and after cystoscopy heart rate (control vs music) were performed (Figures 1 and 2).

For the before vs after cystoscopy heart rate, the music analysis demonstrated the following: a mean difference of – 0.45 (95% CI −4.75 to 3.85), *P* = 0.84, *I^2^ *= 59.4%, Tau^2^ = 8.35 and Cochran’s *Q* = 5.10 (*P* = 0.08). The control analysis demonstrated: a mean difference of – 4.19 (95% CI – 8.70 to 0.32), *P* = 0.07, *I*^2^ = 63.1%, Tau^2^ = 9.57 and Cochran’s *Q* = 5.72 (*P* = 0.06) (Figure 1).

The after cystoscopy heart rate analysis (music vs control) demonstrated the following: a mean difference of – 4.19 (95% CI – 8.70 to 0.32), *P* = 0.07, *I*^2^ = 63.1%, Tau^2^ = 9.57 and Cochran’s *Q* = 5.72 (*P* = 0.06) (Figure 2). Although music had a calming effect with a reduced heart rate, the meta-analysis of the heart rate showed a non-significant reduction in those who underwent cystoscopy with music.

### Individual studies

Zhang *et al*. [7] in 2014 performed a well-designed RCT to investigate the effect of music on male patients undergoing flexible cystoscopy for haematuria and surveillance of bladder cancer. This was a single surgeon series over a 9-month period with 124 patients. The patients in Group 1 (62 patients) were ‘the control group’ and did not listen to music, while the patients in Group 2 (62) could select and listen to their preferred choice of music (classical, Chinese folk, popular music or foreign music) during the procedure, which was standard across both the groups. A VAS was used to evaluate pain [8]. The anxiety levels were calculated according to the STAI scale, which contains 20 self-report items. This has been used to measure tension, worry and apprehension during and after certain procedures [9]. In the 124 patients across the two groups, the mean (SD) pain score on VAS was 2.53 (1.34) in Group 1 (no music) and 1.63 (1.09) in Group 2 (music). The mean (SD) pre- and post-procedural STAI scores for groups 1 and 2 were 41.4 (7.6) and 41.6 (7.9); and 39.4 (6.5) and 34.5 (5.8), respectively. These results were statistically significant (*P* = 0.002) and show that use of music was associated with improved pain and anxiety scores during flexible cystoscopy.

Raheem *et al*. [10] in 2015 conducted a prospective, randomised study to validate the effect of listening to music on perceived anxiety and pain during flexible cystoscopy using VAS and STAI scores within a North American veteran patient population. Over a 2-year period, 137 participants were included (73 in music group and 64 in no music group). The patients in the music group listened to the same excerpt of classical music chosen by the investigators at the time of flexible cystoscopy and no music was played for the patients in the no music group. In that study, pre- and postoperative blood pressure and heart rates were measured for each patient along with other physiological parameters. Over 95% of the patients in the study were men and 13 patients in each group took antidepressant or anti-anxiety medications (18% in music group and 20% in no music group). The median (interquartile range [IQR]) anxiety score in the music group was significantly less than in no music group, at 30 (23–39) vs 35 (28–49). The median delta STAI anxiety scores were also statistically significantly different between the music and no music groups. A similar observation was noted for the VAS scores, which were statistically in favour of the music group. However, the difference in physiological parameters did not reach statistical significance between the music and no music groups.

A similar study by Mirheydar *et al*. [11] in 2015 investigated the effects of music on anxiety and pain relief in a Veteran Affairs population undergoing flexible cystoscopy for surveillance of bladder cancer. In all, 38 male patients were randomly assigned into the no music (24) and music (14) groups, and all patients in the music group listened to the same excerpt of classical music. The median anxiety and pain scores between the two groups were not significantly different. The authors hypothesised that the prevalence of underlying post-traumatic stress disorder (PTSD) and the small number of patients as the reasons why they failed to identify any quantifiable association between music and reduction in pain and anxiety scores [12,13].

Falavolti *et al*. [14] in 2017 investigated the role of listening to music during cystoscopy and biopsy, and whether it decreased pain and discomfort. A total of 100 patients (50 each in the music and no music groups) were included and underwent flexible cystoscopy and cold cup biopsy. After the cystoscopic examination, pain was evaluated by VAS score and the discomfort grade by NRS. Patients in the no music group had significantly higher VAS and NRS scores compared to the music group (*P* < 0.001), although no correlation of listening to music was found for other haemodynamic parameters. The study highlighted the role of music in reducing pain and discomfort for patients undergoing surveillance cystoscopy or biopsy.

## Discussion

### Key findings

The present review shows that in individual studies listening to music significantly reduced anxiety and pain for patients undergoing flexible cystoscopy. Although listening to music had a calming effect, with a reduced heart rate, meta-analysis of heart rate found a non-significant reduction in those who underwent cystoscopy with music. The positive effect was also noted in patients having cystoscopic biopsy, which is more painful than cystoscopy alone.

### Role of music in other surgical and urological procedures

Several studies have confirmed that listening to music is associated with patients experiencing less pain and reduced anxiety during other endoscopic procedures, e.g. bronchoscopy, gastroscopy, colonoscopy and hysteroscopy [15–19]. For example, a RCT in patients undergoing elective colonoscopy concluded that music could decrease the dose of sedation needed [20]. The same has been observed when music was incorporated during suturing of a skin laceration, where the authors highlighted a reduction in doses of sedatives and analgesics [21].

A previous study on the role of music in urology outpatient procedures showed its beneficial effect on decreasing anxiety and pain for prostate biopsy, shockwave lithotripsy, urodynamics, percutaneous nephrostomy, and cystoscopy [5]. It also increased patient satisfaction and patient willingness to undergo the procedure again.

### Effect of music and role of antidepressants/anxiolytics

The role of music in reducing pain and anxiety is not well understood and the mechanism is thought to be complex. It has been postulated that listening to preferred music might provide an emotional distraction leading to reduced pain perception [22]. Functional MRI analysis has highlighted that distraction can increase activation of the cingulo-frontal cortex, the periaqueductal grey, and the posterior thalamus. The authors suggested that this may reduce pain intensity and effect pain-related areas in the brain [23]. Some studies have reported listening to music has been beneficial for analgesia in certain invasive procedures and in cases of chronic pain treatment [17,24,25].

The study by Mirheydar *et al*. [11] included 38 men with prior history of bladder cancer and surveillance cystoscopies. Four patients (29%) in the music group and six (25%) in the no music group were on antidepressant or anti-anxiety medications. The hypothesise for their study not showing an effect of music was that it was possibly due to the increased prevalence of PTSD and generalised anxiety disorder in this subset of patients. Raheem *et al*. [10] in their study investigated 73 patients in the music group and 64 in the no music group. In all, 13 patients (17.8%) in the music group and 13 (20.3%) in the no music group were on antidepressant or anti-anxiety medications. Both studies looked at physiological parameters, such as heart rate and blood pressure, as objective representations of the emotional state. These parameters are influenced by emotional distress or anxiety and patients who take antidepressants and anti-anxiety medications are likely to have biased the results in these studies, potentially diluting the role of music in these patients which would otherwise be more noticeable.

### Choice of music

It might be important to consider the type of music. Most of the participants in the studies were subjected to classical music. Ideally, the patient should be allowed to choose the type of music. However, there seems to be conflicting reports, as some studies have shown that patient-selected music was better and others stating that clinician/researcher selected music was better for improving outcomes [22,26,27]. These studies have analysed factors such as moderate volume, melody, use of percussive instruments and rhythm, factors that may influence patients’ choice of ‘therapeutic music’.

### Strengths and limitations of the study

The strength of our present study is the systematic approach to appraisal of the available literature on the role of music for flexible cystoscopy. The eligible studies had validated tools, such as VAS and STAI scores, to measure the subjective outcomes. Although the music-delivery method was not standardised, Hole *et al*. [28] in their sub-analysis showed that mode of music delivery made little difference to the overall results. Similarly, issues on cost analysis, quality of life, and patient preference were not included.

## Conclusion

The present review has shown that listening to music is associated with reduced anxiety and pain during flexible cystoscopy. It is likely to therefore increase procedural satisfaction and willingness to undergo the procedure again, considering repeated flexible cystoscopy is often needed for surveillance. As music is simple, inexpensive and easily accessible, it should be routinely offered to patients for outpatient and office-based urological procedures.
